# Single-point single-molecule FRAP distinguishes inner and outer nuclear membrane protein distribution

**DOI:** 10.1038/ncomms12562

**Published:** 2016-08-25

**Authors:** Krishna C Mudumbi, Eric C Schirmer, Weidong Yang

**Affiliations:** 1 Department of Biology, Temple University, Philadelphia, Pennsylvania 19122, USA; 2 The Wellcome Trust Centre for Cell Biology, University of Edinburgh, Edinburgh EH9 3BF, UK

## Abstract

The normal distribution of nuclear envelope transmembrane proteins (NETs) is disrupted in several human diseases. NETs are synthesized on the endoplasmic reticulum and then transported from the outer nuclear membrane (ONM) to the inner nuclear membrane (INM). Quantitative determination of the distribution of NETs on the ONM and INM is limited in available approaches, which moreover provide no information about translocation rates in the two membranes. Here we demonstrate a single-point single-molecule FRAP microscopy technique that enables determination of distribution and translocation rates for NETs *in vivo*.

## Abstract (web summary)

Nuclear envelope transmembrane proteins (NETs) can reside in the outer or inner nuclear membrane, but distinguishing which membrane they reside in, and their translocation rate, is technically challenging. Here the authors develop a FRAP-based super-resolution microscopy method to obtain this information for several NETs.

## Graphical abstract

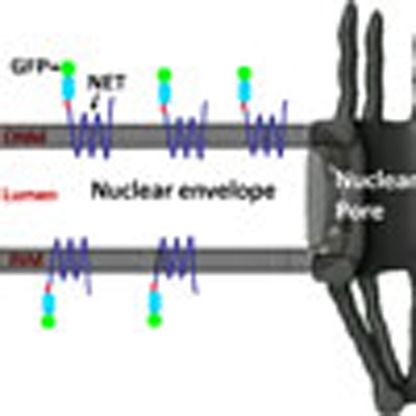

The nuclear envelope (NE) consists of the outer nuclear membrane (ONM) that is contiguous with the endoplasmic reticulum (ER) and the inner nuclear membrane (INM), which is lined with nuclear lamina and faces the nucleoplasm. Both membranes fuse at sites of nuclear pore complex (NPC) insertion. NE transmembrane proteins (NETs) embedded in either the ONM or the INM play crucial roles in both nuclear structure and functions^1^,^2^,^3^,^4^,^5^,^6^,^7^,^8^,^9^. Quantitatively determining the spatial locations of NETs along the NE and translocation rates between the two membranes is needed to fully understand their roles in genome architecture, epigenetics, transcription, splicing, DNA replication, nuclear structure, organization and positioning. Moreover, over a dozen human diseases are associated with mutations and mislocalization of NETs on the NE^10^,^11^,^12^,^13^.

Immunogold-label electron microscopy has been used to determine the localizations of a small set of NETs along the ONM and INM^14^,^15^,^16^. However, it is impractical to apply this labour-intensive approach to the hundreds of NETs now identified^3^. Recently several super-resolution microscopy techniques (STORM, PALM and RESOLFT/STED) have been employed to obtain sub-diffraction images in live cells^17^. Most of these techniques were shown to provide approximately a 50-nm imaging resolution *in vivo*^17^, which render them unlikely to distinguish the real-time localizations of NETs on the INM and ONM, since the two membrane bilayers are separated by a 40-nm perimembrane space^17^.

Fluorescence recovery after photobleaching (FRAP) was developed to mainly study cell membrane diffusion and protein binding^18^. In past years, the technique has been widely applied to study various membrane protein dynamics on the lipid bilayer^19^,^20^,^21^, including the lateral diffusion of NETs on the NE^14^,^16^. Particularly, the technique has been combined with two-photon microscopy to restrict the photobleaching area and provide a better spatiotemporal resolution^22^. Here we have further developed the FRAP technique by adapting a diffraction-limit photobleaching area and recording the recovery of single NETs on the INM and ONM with super-high spatiotemporal resolutions in live cells.

By combining single-point illumination and single-molecule fluorescence recovery after photobleaching (smFRAP), here we show that the spatial localizations of NETs on the INM and ONM are distinguished with a spatial resolution of <10-nm in real-time. Moreover, through measuring the diffusion coefficients and the immobilized fractions of these NETs, we further determine the *in vivo* translocation rates and concentrations of NETs along the ONM and INM. In this paper, several different NETs with unique NE localizations are used to verify and highlight the capabilities of this technique.

## Results

### Single-point smFRAP set-up

**Figure 1:**
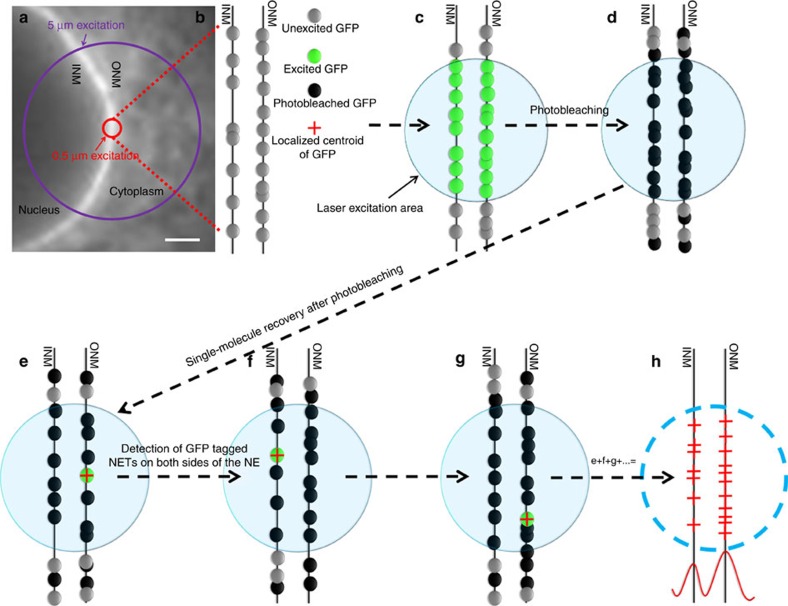
Single-point single-molecule FRAP to study NET distribution along the NE. (**a**) HeLa cell transfected with GFP tagged NET used to visualize the NE. The purple circle indicates the usual 5-μm illumination area used in bulk FRAP experiments and the red circle indicates single-point illumination area up to 0.5 μm used in this study. (**b**) Both the INM and ONM of the NE are studded with NETs fused to GFP. (**c**) Using single-point illumination, a small, 0.5-μm area of the NE is targeted and GFP fused NETs in this area are excited using a high laser power. (**d**) NETs in the laser excitation area, as well as those that diffuse into the area are photobleached. (**e**–**g**) Once the area is completely photobleached, diffusion events of freshly incoming GFP-NETs occur at the single-molecule level and can be precisely localized. (**h**) Localized single-molecule events from the ONM and INM are compiled and the data is fitted with Gaussian functions to determine 2D distribution of NETs along the NE. Scale bar: 1 μm.

In our set-up, the single-point illumination was realized by using a diffraction-limit illumination volume (illumination point spread function) of a 488-nm excitation laser (≈210 nm in the x and y directions and ≈540 nm in *z* direction) generated using a microscope objective with a high numerical aperture. With this single-point illumination at the nuclear equator of live HeLa cells, we conducted the smFRAP measurements of NETs. First, we quickly photobleached GFP-tagged NETs in the illumination area, and then captured individual fluorescent GFP-NETs diffusing into this photobleached area from outside regions with a regulated on-off laser excitation mode. Finally, we reconstructed all detected locations of GFP-NETs to form two-dimensional (2D) super-resolution images (Fig. 1; Supplementary Fig. 1). The combination of single-point illumination and smFRAP allowed us to: (i) generate a laser power with a very high optical density (∼500 kW cm^−2^) to effectively photobleach GFP-NETs in the selected region (Fig. 1a); (ii) adopt a fast detection speed of 2,500 Hz (0.4 ms per frame) to capture GFP-NET molecules in the process of FRAP; (iii) spatially localize GFP-NET molecules with a localization precision of <10 nm (Supplementary Fig. 2); and (iv) treat the NE as relatively straight double bilayers after the full consideration of membrane curvature and maximally reduce the possible photodamage in live cells because of the very small illumination volume (Supplementary Fig. 3). With this set-up, each single-molecule video was recorded for 30 s then filtered by signal photons of single protein molecules. Ten such single-molecule videos from a live cell yielded ∼10,000 spatial locations of NETs in the NE with a total microscope and analysis time of <30 min. Such measurements were repeated in ten different live cells. Finally, the resultant data was used to quantitatively determine the spatial distribution of NETs along the INM and ONM in live cells.

### Concentration ratio of LBR on the INM and ONM

**Figure 2:**
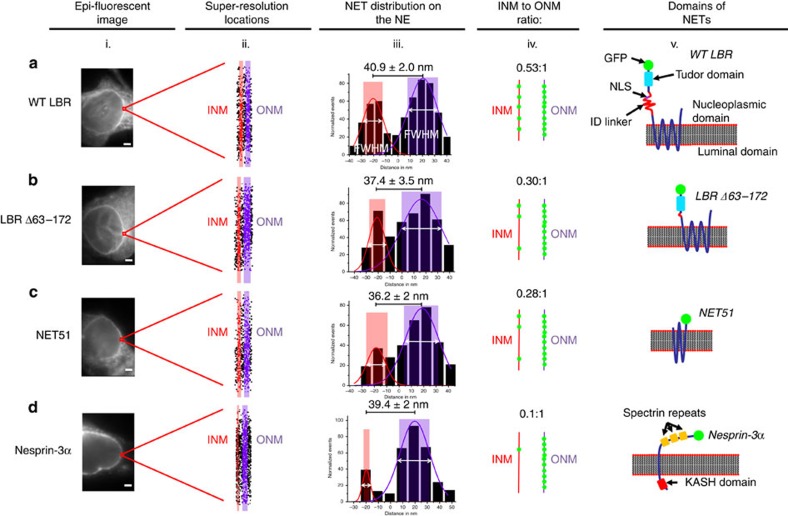
Super-resolution imaging and distribution of various NETs. Wild-type LBR (WT LBR) (**a**). LBR Δ63–172 (**b**), NET51 (**c**) and Nesprin-3α (**d**). (i) Epi-fluorescent image of the NE of a HeLa cell transfected to express the NET of interest. The area that was photobleached and studied is boxed in red. (ii) Super-resolution image of the NE with the INM shown in red and the ONM shown in purple. To obtain these locations, typically ten 30-s single-molecule videos from a live cell yielded ∼10,000 spatial locations of NETs in the NE. Such measurements were repeated in ten different live cells. (iii) Two peak Gaussian fittings of the points collected from the NE showing the distribution of NETs along the NE. The INM to ONM ratio was determined by using the integrated area under the fitted curves. The shaded regions represent the width of the INM and ONM as determined by the full width at half maximum (FWHM) as determined by the fitting. (iv) Approximate concentration ratios of NET's distribution (pre-corrected) along the INM (red) and ONM (purple). The corrected ratios can be found in Table 1. (v) Illustrative representation of the GFP fused NETs used in this study. Scale bar, 1 μm.

The localization of GFP-tagged wild-type lamin B receptor (WT LBR) was first determined in live HeLa cells. LBR is an important INM protein that interacts with chromatin and lamins. With the single-point smFRAP microscopy setup, we obtained ∼8,000 locations of WT LBR on the NE and the histogram of these locations across the NE revealed two major peaks with a distance of 40.9±2.0 nm, which agrees well with the 40-nm perimembrane space between the INM and ONM (6). The integrated areas of these two peaks further revealed that WT LBR localizes on these two layers with an INM:ONM concentration ratio of 0.53:1 (Fig. 2a; Supplementary Fig. 4).

Since the concentration ratio is intrinsically tied to the diffusion coefficient and the immobile fraction of NETs that have not been included in the above determined concentration ratio, corrections must be made to determine the actual distribution of NETs along the NE. Therefore, based on the single-molecule trajectories of WT LBR molecules as they diffused along the ONM and INM, we first determined the diffusion coefficient (*D*) of these proteins in each membrane using their mean squared displacements or the frequency distribution (Methods). WT LBR on the ONM possesses a slightly bigger diffusion coefficient than that on the INM, as expected due to its INM binding partners (Table 1). Second, to correlate the determined diffusion coefficients with the actual concentrations of WT LBR on the INM (*C*_INM_) and ONM (*C*_ONM_), we developed the following equations:


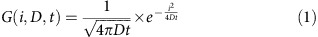



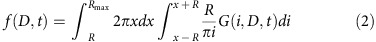



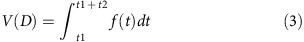



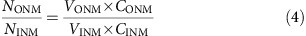


Where *G (i, D, t)* represents the probability of finding a randomly diffusing particle at location *i* after diffusion with a diffusion constant of *D* within time *t*; *f (D, t)* refers to the probability of observing the particles moving into the detection area in two dimensions from the entire area; *V*(*D*) is the total area that a particle covered from *t1* to *t1*+*t*2 s; and *V* (either ONM or INM) is calculated from the previous function and *N* (ONM or INM) is determined from the original INM:ONM ratio. Supplementary Fig. 5 provides a visual representation of these calculations. By considering the effect of diffusion coefficients, the corrected concentration ratio of WT LBR on the INM and ONM is 0.58:1 (Table 1). Moreover, from the bulk FRAP curves (Supplementary Table 1 and Supplementary Fig. 6), the immobilized fraction of NETs on the INM can be obtained (using the formulae included in the figure caption of Supplementary Fig. 6). For WT LBR, since previous experiments indicate a negligible immobile fraction on the ONM/ER^14^ (and verified in Supplementary Fig. 6), the immobile fraction can fully be ascribed to the INM, and this was measured at ∼81% (Table 1). This number, along with the diffusion-based corrected concentration ratio, was used to determine that the actual INM to ONM ratio of WT LBR is 3.1:1 in live cells (Table 1), which agrees very well with an INM:ONM concentration ratio of 3:1 determined by immunogold-label electron microscopy counting of 440 particles^16^.

### Different NETs possess distinct INM:ONM concentration ratios

Next, following the same experimental protocol, the distribution and concentration of NET51, expected to lack or have unknown INM interactions because of its short nucleoplasmic domain, nesprin-3α, expected only in the ONM, and a mutant of LBR (LBRΔ63–172) that lacks its mapped lamin and chromatin binding sites and predicted NLSs^23^,^24^ on the NE, were determined. In contrast to WT LBR, LBRΔ63–172 was found on the INM and ONM with a diffusion-based corrected concentration ratio of 0.30:1, consistent with a loss of INM binding sites. This is similar to the ratio of 0.29:1 obtained for NET51 that was expected to have no INM binding sites (Fig. 2b,c). The low INM:ONM ratio for NET51 is further supported by the electron microscopy study done by Zuleger *et al*.^16^. Finally, nesprin-3α, a NET that localizes mainly on the ONM, was used as a control to determine whether single-point smFRAP can distinguish between NETs that translocate into the INM compared with those that localize mainly on the ONM. Figure 2d clearly shows that, as expected, nesprin-3α localizes almost completely on the ONM with a diffusion-based corrected ratio of 0.10:1.

Besides the corrections based on the diffusion coefficients, the immobilized fraction of LBRΔ63–172, NET51, and nesprin-3α were measured as well (Supplementary Fig. 6 and supplementary Table 1). When compared with WT LBR, the immobilized fraction in the INM of LBRΔ63–172 reduced from ∼81 to ∼72% (Table 1). The reduction but not complete loss of the LBR immobilized fraction is not surprising. Even though INM-specific binding domains of the LBR protein were deleted to create the LBRΔ63–172 mutant, the tudor domain on the N terminus is still present (Fig. 2b—v), and there is a lesser chromatin binding activity in the C terminus that is also nucleoplasmic. NET51 had a ∼38% INM immobilized fraction, and nesprin-3α had a ∼61% immobilized fraction specifically on the ONM. When the immobilized fractions were taken into account together with the diffusion-based corrected concentration ratios, the final INM:ONM concentrations ratios for LBRΔ63–172, NET51, and nesprin-3α were calculated to be 1.10:1, 0.47:1, and 0.04:1, respectively. This is consistent with the loss of INM binding reducing the INM:ONM ratio for the LBR mutant and the high ONM immobile fraction for nesprin-3α further decreasing its INM:ONM ratio (Table 1).

## Discussion

Using this simple technique, one can not only determine if a NET transports into the nucleus, but also its distribution along the INM and ONM within 30 min with a precision of <10 nm. Also, this technique can be used on NETs tagged with the simple and commonly found GFP tag, as opposed to a specialized tag, such as photoactivatable GFP. Furthermore, this method is done *in vivo* so that almost no cellular functions are disrupted and the actual location of the protein can be observed in the natural cellular environment.

In addition, this technique can also be used to determine the translocation rates (TR) of various NETs across the NE. If the total concentration of a NET on the NE is known, together with the concentration ratio of NETs determined here, a few formulae can be used to determine the translocation rate of NETs from the ONM to the INM as follows:


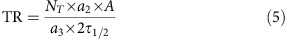



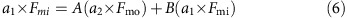



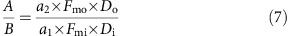


Where *N*_*T*_ is the total NET molecules, *a*_1_, *a*_2_ and *a*_*3*_ are the INM value, the ONM value and the added INM and ONM value from the INM:ONM ratio, respectively (*a*_1_=3.1, *a*_2_=1 and *a*_*3*_=3.1+1 in the case of WT LBR). The variable *A* represents the fraction of NETs in the ONM that translocate into the INM after FRAP experiments and *B* is the fraction of NETs in the INM that diffuse into the photobleached area after FRAP. *F*_mi_ is the mobile fraction on the INM, *F*_mo_ is the mobile fraction on the ONM. *D*_o_ and *D*_i_ are the diffusion coefficients on the ONM and INM respectively as determined by single-molecule experiments, and finally, *τ*_½_ is the time it takes for half the fluorescence recovery during FRAP experiments (Supplementary Table 1). Using LBR as an example, with ∼150,000 LBR molecules and about 2,000 NPCs per cell^25^,^26^, the translocation rate from ONM to INM is about 5.4 molecules per min per NPC.

Finally, based on the immobilized fraction and the diffusion-based distribution concentrations, the single-point smFRAP technique also provides putative information about possible interactions on either the ONM or INM face of the NE for uncharacterized NETs. By studying the distribution ratios and immobilized fractions of WT LBR, LBRΔ63–172, NET51 and nesprin-3α, a putative inference about interactions can be made. For example, even though WT LBR and nesprin-3α have similar immobilized fractions as determined by conventional FRAP, by studying the distribution ratios provided by single-point smFRAP, it can be determined that WT LBR has binding sites that require it to be on the INM for interactions with its binding partners. Nesprin-3α, however, has a higher distribution on the ONM indicating that it interacts with binding partners that are located either in the cytosol or with the luminal domains of proteins located on the INM.

## Methods

### Tissue culture and transfection

HeLa cells were grown in DMEM, high glucose, GlutaMAX Supplement (Life Technologies), 10% fetal bovine serum (Fisher Scientific), 1% penicillin-streptomycin (Thermo Fisher). Nesprin-3α, WT and mutant LBR were cloned into the pEGFP-C3 vector, and NET51 into the pEGFP-N2 vector. DMEM with 1% penicillin-streptomycin and no fetal bovine serum was used for transfection with TransIT-LT1 Transfection Reagent (Mirus Bio) using the manufacturer's protocol. Cells were incubated with pre warmed (37 °C) transport buffer (20 mM HEPES, 110 mM KOAc, 5 mM NaOAc, 2 mM MgOAc, 1 mM EGTA, pH adjusted to 7.3 with HCl) for 45 min before either single-point smFRAP or bulk FRAP experiments. Measurements on the microscope were completed within 30 min to ensure that the cells are monitored in near physiological conditions.

### Single-point smFRAP microscopy

Transfected HeLa cells were imaged with an Olympus IX81 microscope equipped with a 1.4 numerical aperture × 100 oil-immersion objective (UPLSAPO 100XO, Olympus) and with an on-chip multiplication gain CCD camera (Cascade 128+, Roper Scientific). A 50-mW solid-state 488-nm laser (Obis) was used to excite the GFP tagged NETs. Epi-fluorescent imaging was performed using a mercury lamp with GFP filter set-up. The following filters were used: dichroic filter (Di01-R405/488/561/635-25x36, Semrock) and an emission filter (NF01-405/488/561/635-25X5.0, Semrock), two neutral density filters (Newport). A Newport optical chopper was used to generate an on-off mode of laser excitation. For data acquisition and processing, the Slidebook software package (Intelligent Imaging Innovations) was used.

### Bulk FRAP by using confocal microscopy

Bulk FRAP experiments were performed by using a Leica DM IRE2 confocal microscope running TCS SL software. First, five pre-bleach images were taken and averaged to obtain the initial fluorescence intensity value. Then, photobleaching was performed with an argon laser (488-nm laser line) for about 5 s to bleach an area of ∼5 μm^2^. Fluorescence recovery was measured every 5 s until the fluorescence reaches the plateau stage. Finally, image-induced photobleaching was corrected by normalizing to the time-course decay of fluorescence in non-bleached areas by using the ImageJ plug-in FRAP Profiler.

### Determination of diffusion coefficient

Two complementary approaches have been used to determine the diffusion coefficients for NET proteins. First, if single-molecule trajectories of a protein molecule consist of multiple frames (>6), we used the typical mean square displacement (MSD, MSD=4*Dt* for 2D trajectories) approach. Second, if there are at least two, but less than six consecutive frames obtained, we utilized the frequency distribution probability function 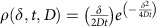 (refs 27, 28), where *δ, t* and *D* are the displacement between consecutive frames, the interval time and the diffusion coefficient respectively. Approximately 50 single-molecule trajectories were collected and processed by utilizing the first approach and >500 events were used by following the second approach. Finally, an averaged diffusion coefficient was determined for each NET.

### Localization precisions of isolated fluorescent spots

The localization precision for single fluorescent molecules was defined as how precisely the central point of each detected fluorescent diffraction-limited spot was determined. Typically, for immobilized molecules, the fluorescent spot is fitted to a 2D symmetrical Gaussian function, and the localization precision is determined by the s.d. of multiple measurements of the central point. However, for moving molecules, the influence of particle motion during image acquisition should be considered in the determination of localization precision. In detail, the localization precision for moving substrates (σ) was determined by an algorithm of 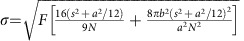, where *F* is equal to 2, *N* is the number of collected photons, *a* is the effective pixel size of the detector, *b* is the s.d. of the background in photons per pixel, and 
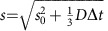
, *s*_*0*_ is the s.d. of the point spread function in the focal plane, *D* is the diffusion coefficient of substrate on the membrane of interest (INM or ONM) and *Δt* is the image acquisition time^29^,^30^,^31^,^32^.

In our experiments, we spatially localized and superposed targeted molecules with >2,000 signal photons and in-focus Gaussian widths (0.5–1.0 pixel, corresponding to single GFP molecules locating in the focal plane). Thus, the localization precision is determined to be <10 nm based on the above equations and the parameters determined experimentally (*N*>2,000, *a*=240 nm, *b*≈2, *s*_*0*_=150±50 nm, *D* is in the range of 1–3 μm^2^ s^−1^ for the tested substrates), as shown in Supplementary Fig. 2.

### Data analysis

Single-molecule videos were recorded using Slidebook (Intelligent Imaging Innovations) and then analyzed with the ImageJ plugin ThunderSTORM (zitmen.github.io/thunderstorm/) and the raw data was filtered with a high signal to noise ratio (SNR) and the average *x* and *y* pixel positions were determined to select the region of interest for analysis with the GLIMPSE software package (courtesy of the Gelles Lab). Raw data were run through GLIMPSE and selected for a high SNR to ensure that single molecules in the focal plane are selected and analysed. The resultant data were then fit with a single Gaussian distribution to remove more background noise and that data were then fit with a two peak Gaussian to determine the distribution of NETs along the NE (Supplementary Fig. 4). Finally, the FWHM of each peak was used to determine the range of NET distribution on each of the two membranes, which was used when studying single-molecule trajectories for determining the diffusion coefficient.

### Statistical analysis

Both single-molecule and bulk FRAP measurements were repeated in at least 10 different live cells. Experimental measurements are reported as mean±s.e.m., unless otherwise noted.

### Data availability

The data that support the findings of this study are available on request from the corresponding author (W.Y.).

## Additional information

**How to cite this article:** Mudumbi, K. C. *et al*. Single-point single-molecule FRAP distinguishes inner and outer nuclear membrane protein distribution. *Nat. Commun.* 7:12562 doi: 10.1038/ncomms12562 (2016).

## Supplementary Material

Supplementary Information Supplementary Figures 1-6, Supplementary Table 1

## Figures and Tables

**Table 1:** Distribution of NET substrates on the ONM and INM.

**Protein**	**Single-molecule based concentration ratio (INM:ONM)**	**Diffusion coefficient on ONM (μm**^**2**^** s**^**−1**^**)**	**Diffusion coefficient on INM (μm**^**2**^** s**^**−1**^**)**	**Diffusion based corrected concentration ratio (INM:ONM)**	**Immobilized fraction**	**Final Concentration ratio (INM:ONM)**
WT LBR	0.53:1	2.6±0.8	1.9±0.6	0.58:1	61±6% (overall)	3.1:1
					81±6% (INM)	
LBRΔ63–172	0.30:1	1.1±0.3	1.0±0.4	0.30:1	37±8% (overall)	1.1:1
					72±8% (INM)	
NET51	0.28:1	2.0±0.6	1.8±0.7	0.29:1	12±8% (overall)	0.47:1
					38±8% (INM)	
Nesprin-3α	0.10:1	1.2±0.5	0.9±0.4	0.10:1	61±7% (overall)	0.04:1
					61±7% (ONM)	

FRAP, fluorescence recovery after photobleaching; INM, inner nuclear membrane; NE, nuclear envelope; NET, NE transmembrane proteins; ONM, outer nuclear membrane; WT LBR, wild-type lamin B receptor.

In the column of ‘Immobilized fraction', ‘overall' refers the immobilized fraction of NETs on the NE and ‘INM' represents the immobilized fraction of NETs on the INM (using the formulae included in the figure caption of Supplementary Fig. 6). Both single-molecule and bulk FRAP measurements were repeated in at least ten different live cells. Data are mean±s.e. of the mean.

## References

[b1] Arib G. & Akhtar A. Multiple facets of nuclear periphery in gene expression control. Curr. Opin. Cell Biol. 23, 346–353 (2011). 21242077 10.1016/j.ceb.2010.12.005

[b2] Burns L. T. & Wente S. R. Trafficking to uncharted territory of the nuclear envelope. Curr. Opin. Cell Biol. 24, 341–349 (2012). 22326668 10.1016/j.ceb.2012.01.009 PMC3518394

[b3] de las Heras J. I. . Tissue specificity in the nuclear envelope supports its functional complexity. Nucleus 4, 460–477 (2013). 24213376 10.4161/nucl.26872 PMC3925691

[b4] Gruenbaum Y., Margalit A., Goldman R. D., Shumaker D. K. & Wilson K. L. The nuclear lamina comes of age. Nat. Rev. Mol. Cell Biol. 6, 21–31 (2005). 15688064 10.1038/nrm1550

[b5] Heessen S. & Fornerod M. The inner nuclear envelope as a transcription factor resting place. EMBO Rep. 8, 914–919 (2007). 17906672 10.1038/sj.embor.7401075 PMC2002563

[b6] Hetzer M. W. & Wente S. R. Border control at the nucleus: biogenesis and organization of the nuclear membrane and pore complexes. Dev. Cell 17, 606–616 (2009). 19922866 10.1016/j.devcel.2009.10.007 PMC3746554

[b7] Wilson K. L. & Foisner R. Lamin-binding proteins. Cold Spring Harb. Perspect. Biol. 2, a000554 (2010). 20452940 10.1101/cshperspect.a000554 PMC2845209

[b8] Zuleger N., Korfali N. & Schirmer E. Inner nuclear membrane protein transport is mediated by multiple mechanisms. Biochem. Soc. Trans. 36, 1373 (2008). 19021558 10.1042/BST0361373

[b9] Zuleger N., Kerr A. R. & Schirmer E. C. Many mechanisms, one entrance: membrane protein translocation into the nucleus. Cell Mol. Life Sci. 69, 2205–2216 (2012). 22327555 10.1007/s00018-012-0929-1 PMC11114554

[b10] Dauer W. T. & Worman H. J. The nuclear envelope as a signaling node in development and disease. Dev. Cell 17, 626–638 (2009). 19922868 10.1016/j.devcel.2009.10.016

[b11] Méndez-López I. & Worman H. J. Inner nuclear membrane proteins: impact on human disease. Chromosoma 121, 153–167 (2012). 22307332 10.1007/s00412-012-0360-2

[b12] Schreiber K. H. & Kennedy B. K. When lamins go bad: nuclear structures and disease. Cell 152, 1365–1375 (2013). 23498943 10.1016/j.cell.2013.02.015 PMC3706202

[b13] Worman H. J. & Dauer W. T. The nuclear envelope: an intriguing focal point for neurogenetic disease. Neurotherapeutics 11, 764–772 (2014). 25119890 10.1007/s13311-014-0296-8 PMC4391386

[b14] Ellenberg J. . Nuclear membrane dynamics and reassembly in living cells: targeting of an inner nuclear membrane protein in interphase and mitosis. J. Cell Biol. 138, 1193–1206 (1997). 9298976 10.1083/jcb.138.6.1193 PMC2132565

[b15] Wilhelmsen K. . Nesprin-3, a novel outer nuclear membrane protein, associates with the cytoskeletal linker protein plectin. J. Cell Biol. 171, 799–810 (2005). 16330710 10.1083/jcb.200506083 PMC2171291

[b16] Zuleger N. . System analysis shows distinct mechanisms and common principles of nuclear envelope protein dynamics. J. Cell Biol. 193, 109–123 (2011). 21444689 10.1083/jcb.201009068 PMC3082195

[b17] Godin A. G., Lounis B. & Cognet L. Super-resolution microscopy approaches for live cell imaging. Biophys. J. 107, 1777–1784 (2014). 25418158 10.1016/j.bpj.2014.08.028 PMC4213717

[b18] Axelrod D., Koppel D., Schlessinger J., Elson E. & Webb W. Mobility measurement by analysis of fluorescence photobleaching recovery kinetics. Biophys. J. 16, 1055 (1976). 786399 10.1016/S0006-3495(76)85755-4 PMC1334945

[b19] Fritzsche M. & Charras G. Dissecting protein reaction dynamics in living cells by fluorescence recovery after photobleaching. Nat. Protoc. 10, 660–680 (2015). 25837418 10.1038/nprot.2015.042

[b20] Sprague B. L. & McNally J. G. FRAP analysis of binding: proper and fitting. Trends Cell Biol. 15, 84–91 (2005). 15695095 10.1016/j.tcb.2004.12.001

[b21] Sprague B. L., Pego R. L., Stavreva D. A. & McNally J. G. Analysis of binding reactions by fluorescence recovery after photobleaching. Biophys. J. 86, 3473–3495 (2004). 15189848 10.1529/biophysj.103.026765 PMC1304253

[b22] Coscoy S. . Molecular analysis of microscopic ezrin dynamics by two-photon FRAP. Proc. Natl Acad. Sci. USA 99, 12813–12818 (2002). 12271120 10.1073/pnas.192084599 PMC130542

[b23] Ye Q. & Worman H. J. Primary structure analysis and lamin B and DNA binding of human LBR, an integral protein of the nuclear envelope inner membrane. J. Biol. Chem. 269, 11306–11311 (1994). 8157662

[b24] Ye Q. & Worman H. J. Interaction between an integral protein of the nuclear envelope inner membrane and human chromodomain proteins homologous to Drosophila HP1. J. Biol. Chem. 271, 14653–14656 (1996). 8663349 10.1074/jbc.271.25.14653

[b25] Schwanhäusser B. . Global quantification of mammalian gene expression control. Nature 473, 337–342 (2011). 21593866 10.1038/nature10098

[b26] Maul G. & Deaven L. Quantitative determination of nuclear pore complexes in cycling cells with differing DNA content. J. Cell Biol. 73, 748–760 (1977). 406262 10.1083/jcb.73.3.748 PMC2111421

[b27] Kues T., Peters R. & Kubitscheck U. Visualization and tracking of single protein molecules in the cell nucleus. Biophys. J. 80, 2954–2967 (2001). 11371468 10.1016/S0006-3495(01)76261-3 PMC1301479

[b28] Smith P. R., Morrison I. E. G., Wilson K. M., Fernandez N. & Cherry R. J. Systems analysis of Ran transport. Biophys. J. 76, 3331–3344 (1999). 10354459 10.1016/S0006-3495(99)77486-2 PMC1300303

[b29] Mortensen K. I., Churchman L. S., Spudich J. A. & Flyvbjerg H. Optimized localization analysis for single-molecule tracking and super-resolution microscopy. Nat. Methods 7, 377 (2010). 20364147 10.1038/nmeth.1447 PMC3127582

[b30] Quan T. W., Zeng S. Q. & Huang Z. L. Localization capability and limitation of electron-multiplying charge-coupled, scientific complementary metal-oxide semiconductor, and charge-coupled devices for superresolution imaging. J. Biomed. Opt. 15, 066005 (2010). 21198179 10.1117/1.3505017

[b31] Robbins M. S. & Hadwen B. J. The noise performance of electron multiplying charge-coupled devices. IEEE Trans. Electron Devices 50, 1227 (2003).

[b32] Deschout H., Neyts K. & Braeckmans K. The influence of movement on the localization precision of sub-resolution particles in fluorescence microscopy. J. Biophoton. 5, 97 (2012). 10.1002/jbio.201100078 22083848

