# Supplementary material for: Single-point single-molecule FRAP distinguishes inner and outer nuclear membrane protein distribution
Source: Nat Commun. 2016 Aug 25;7:12562. doi: 10.1038/ncomms12562 (PMC5007294; doi:10.1038/ncomms12562)
Supplement: Supplementary Information — Supplementary Figures 1-6, Supplementary Table 1 [file ncomms12562-s1.pdf]

SUPPLEMENTARY INFORMATION

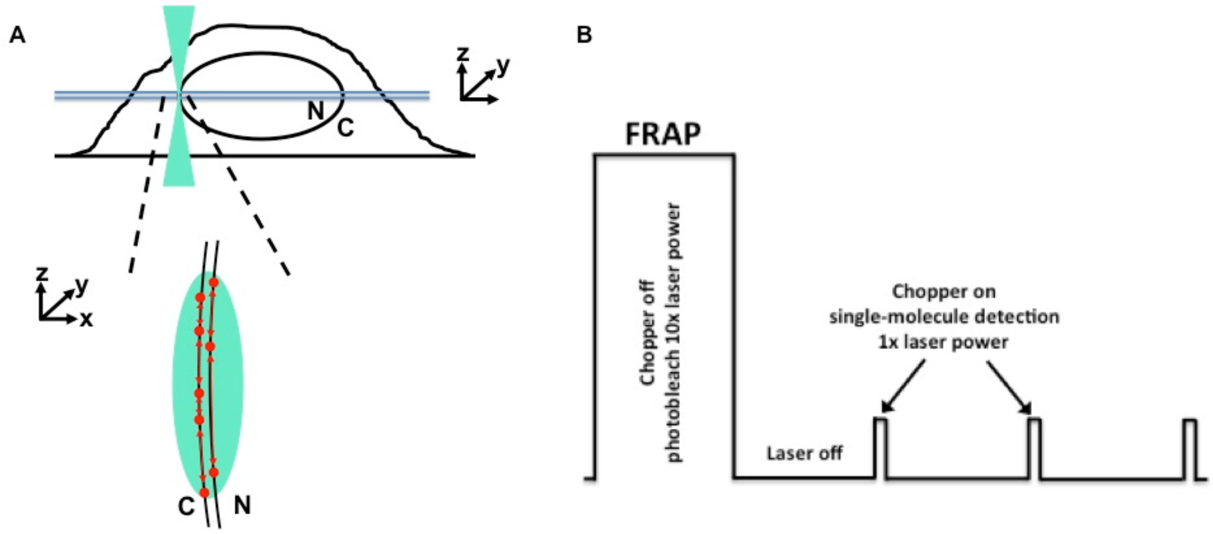

Supplementary Figure 1. Single-point single-molecule FRAP used to detect transmembrane proteins on the NE. (A) Imaging of single-molecule events of NETs along the NE by single-point smFRAP microscopy. The focal plan is between the two light blue lines. C, cytoplasm; N, nucleus. (B) Ten-fold different laser powers were used to photobleach and detect in the experiments. An optical chopper was used regulate the laser to have an on-off mode. The longer off time allowed GFP-NETs outside the photobleached area to have enough time to diffuse into the detection area.

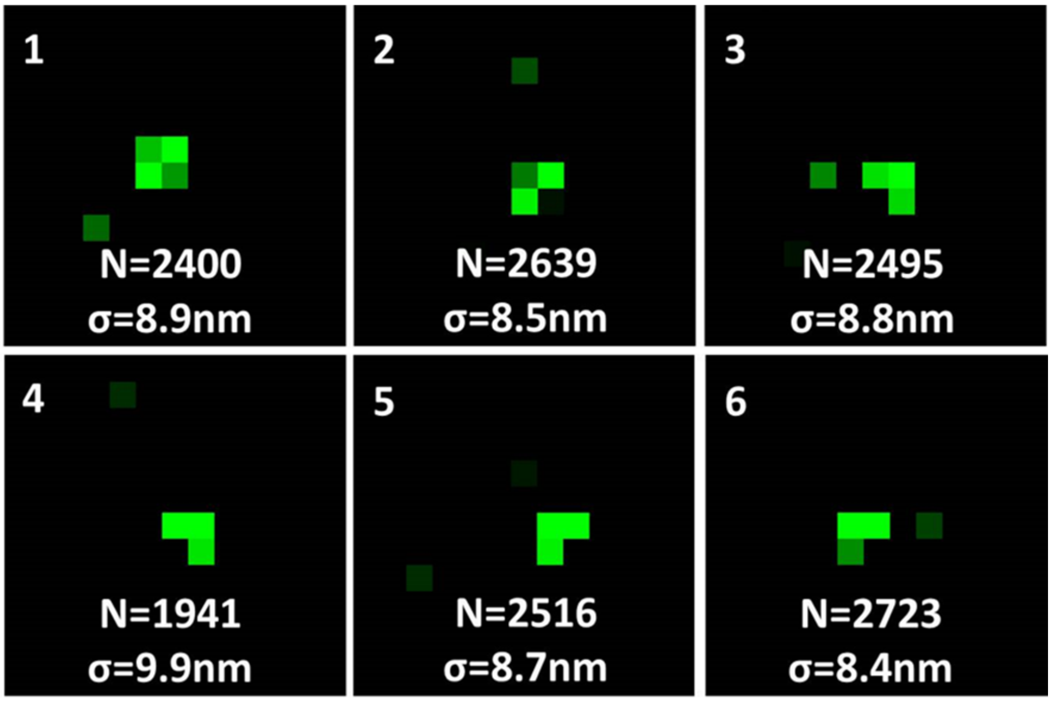

Frame	Total Intensity	Photons	Precision (nm)
1	181090	2400	8.9
2	199158	2693	8.5
3	188263	2495	8.8
4	146507	1941	9.9
5	189832	2516	8.7
6	205467	2723	8.4
S.D. of background	1105	2/pixel	-

Supplementary Figure 2. Typical photons and localization precision for single LBR on NE. Six typical single-molecule images of LBR were shown as examples and the total intensity, the total photons and the standard derivation (S.D.) of background in photon per pixel were listed as well. Based on these information and the equations in the "Localization Precisions of Isolated Fluorescent Spots" of Methods section, the localization precision of single LBR molecules were determined to be < 10 nm. Pixel size: 240 nm.

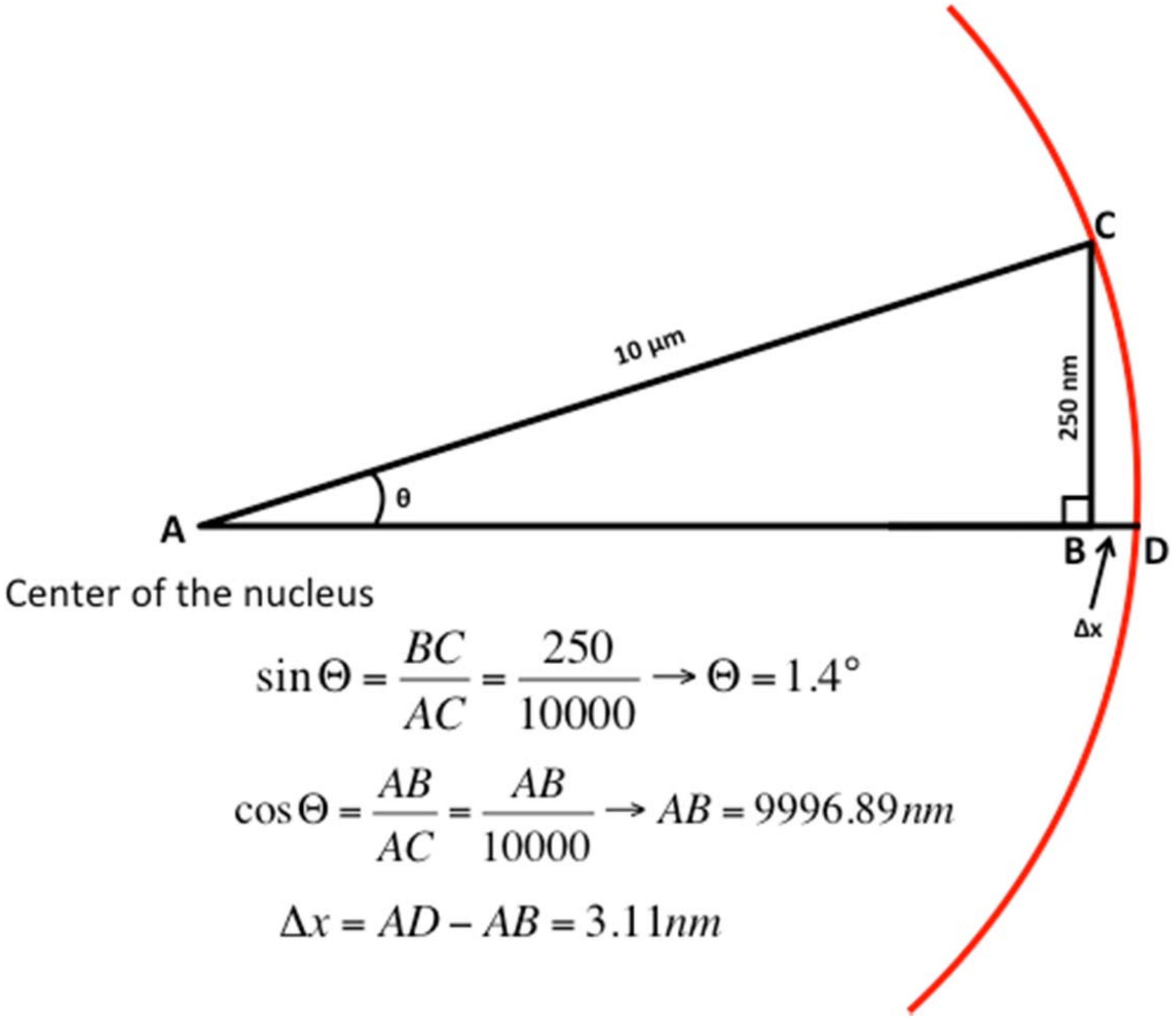

$$\sin\Theta = \frac{BC}{AC} = \frac{250}{10000} \rightarrow \Theta = 1.4^\circ$$

$$\cos\Theta = \frac{AB}{AC} = \frac{AB}{10000} \rightarrow AB = 9996.89nm$$

$$\Delta x = AD - AB = 3.11nm$$

Supplementary Figure 3. Calculation for membrane curvature. Point A is the center of the nucleus of a HeLa cell, which is assumed to have an average radius of about 10 μm (line AC and AD), and the membrane is represented by the curved red line. BC is half the PSF of the illumination area, 250 nm. From this, it can be calculated that the membrane curves only about 1.4° (Θ, arc CD), which would cause a slight error (Δx) of ~ 3.11 nm at the very edge of PSF of the illumination area (point C) from the center (point D). The error was fully considered in the correction of 2D super-resolution spatial locations of GFP-NETs.

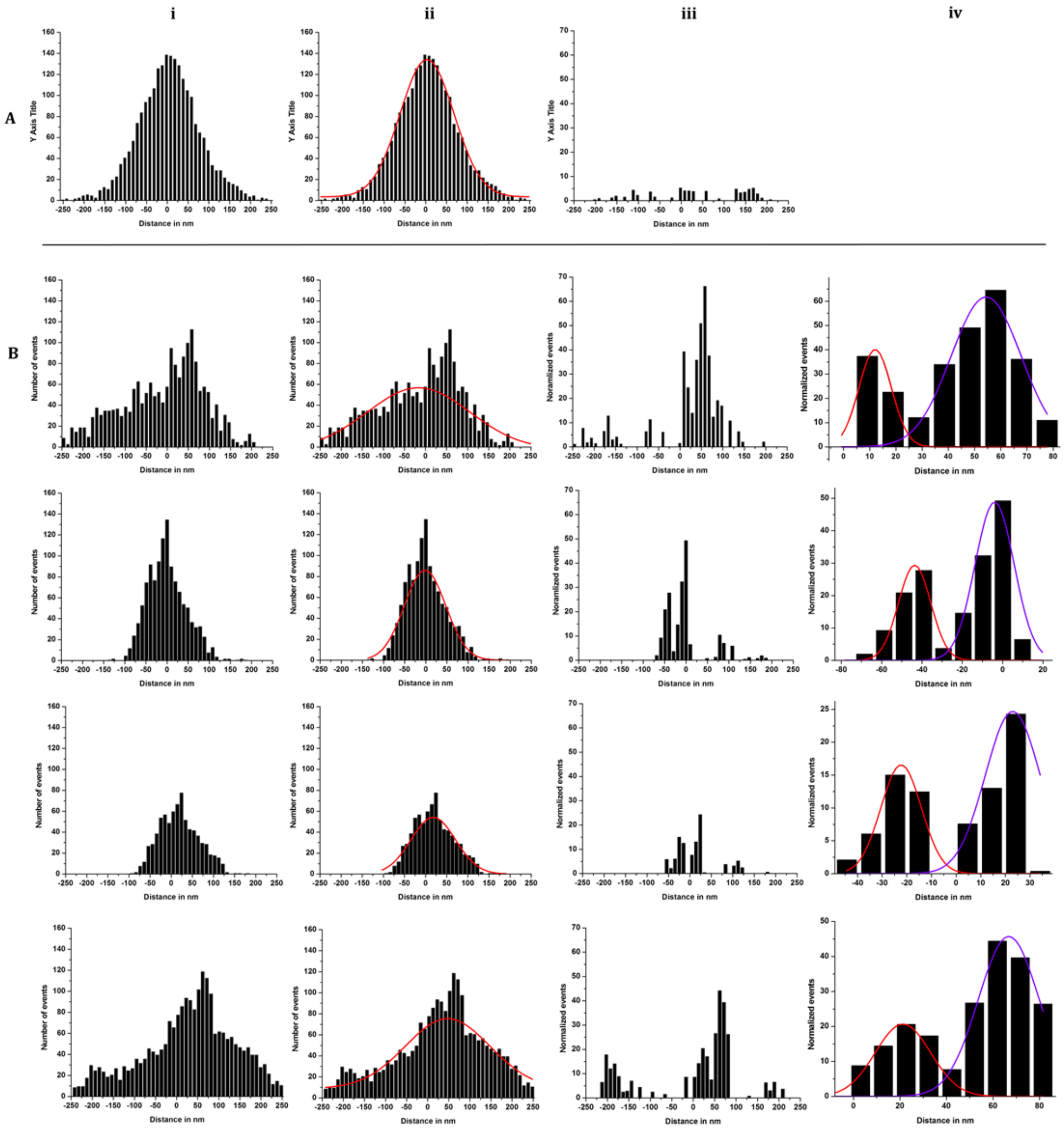

Supplementary Figure 4. Background subtraction from raw data. A) Data from a control experiment of GFP molecules freely diffusing in the cytosol. B) Representative data from various experiments with NETs on NE in HeLa cells. Here we show the process of extracting super-resolution locations of INM and ONM from raw data, by listing all the typical cases we observed in our experiments. i) Histograms were prepared from the raw data. Different from the control experiment, the distributions of NETs reveal obvious peaks above the normal distribution. ii) The background (excluding the data within ±40 nm of the highest peak) was then fit with a single Gaussian function (show in red). iii) Next, the fitted Gaussian distribution was subtracted from the raw data to generate a histogram of the normalized data showing two clear maximum peaks. While, for the control experiment, only small random residues remained after subtraction. iv) These two maximum peaks were then fit with a two-peak Gaussian function to determine the localization of NETs on the NE (INM shown in red and ONM shown in purple).

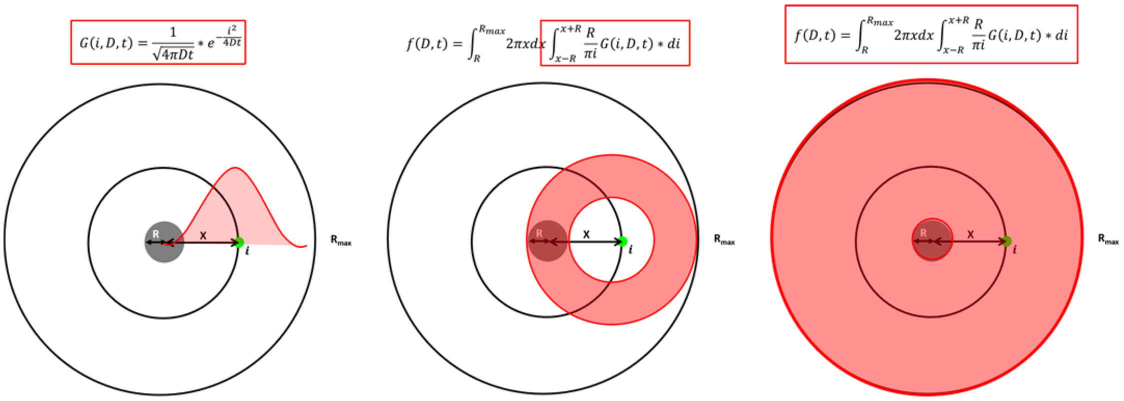

Supplementary Figure 5. Method used to correct the ONM:INM ratios by including the effect of molecular diffusion coefficient as determined by single-molecule trajectories. This calculation takes into account the differing two dimensional diffusion coefficients of transmembrane proteins along the nuclear envelope of the cell as they enter the detection area (shown in gray), and corrects the distribution ratio to reflect the actual transmembrane protein concentrations along the nuclear envelope. The outer ring (R_{max}) represents the entire circumference of the nuclear envelope and the detected molecule (shown in green) can come from any locations with the distance X from the center of the photobleached area (indicated by the second inner ring). (A) G (i, D, t) represents the probability of finding a randomly diffusing particle at location i after diffusion with a diffusion constant of D within time t. (B) The probability that molecules starting at i (shown in green) eventually diffuse into the detection area (gray). (C) f (D, t) refers to the probability of observing the particles moving into the detection area in two dimensions from the entire area (R_{max}).

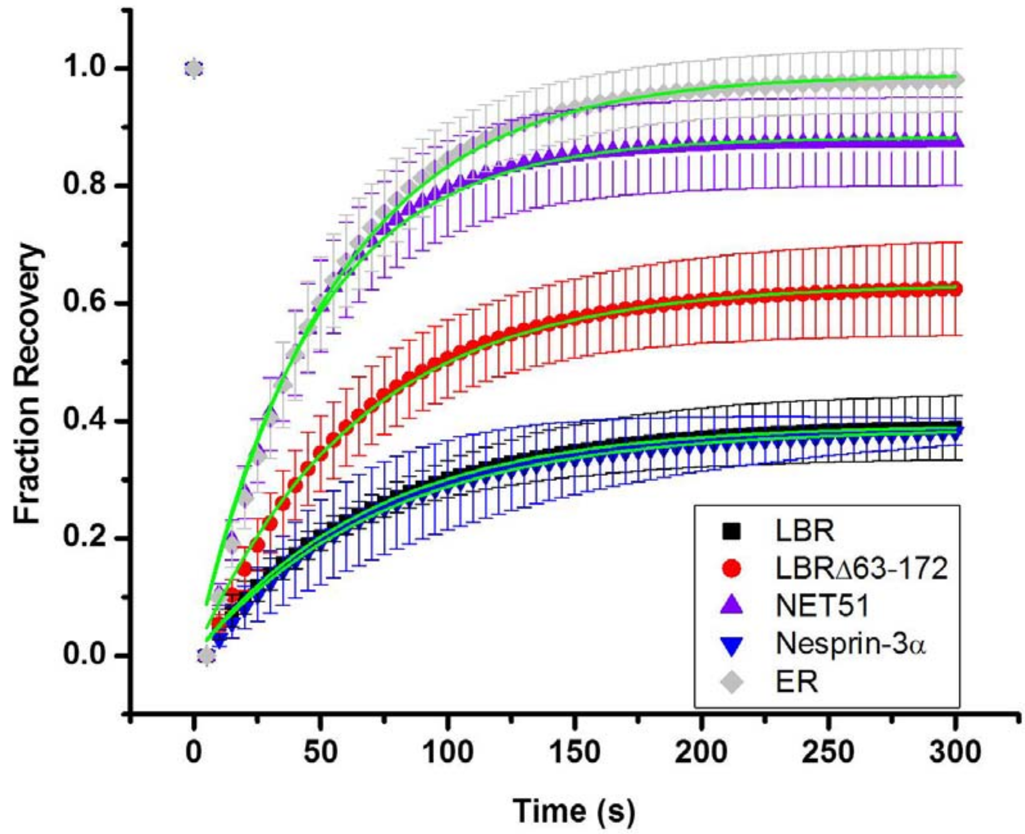

Supplementary Figure 6. FRAP curves for wt LBR, LBRΔ63-172, NET51, and nesprin-3α. Photobleaching was performed with an argon laser (488-nm laser line) for about 5 seconds to bleach an area of approximately 5 μm². Fluorescence recovery was measured every 5 seconds till the fluorescence reaches the plateau stage. Following the typical process, the ER was used as a control to verify the mobility of NETs on the ER/ONM. Exponential functions have been applied to fit all the measured curves averaged on ten different NEs. The following two formulae were used to calculate the immobilized fraction of NETs on the INM (except for nesprin-3α which has an immobile fraction mainly on the ONM): $N_{ONM} + (1-x) * N_{INM} = F_m(N_{ONM} + N_{INM})$ and $\frac{N_{ONM}}{(1-x)*N_{INM}} = R$. Here, the actual number of the NET on the ONM is N_{ONM} and on the INM is N_{INM}. For most NETs, previous measurements suggested that their ONM mobile fraction is almost 100% and here the INM immobile fraction is defined as x. F_{m} is the mobile fraction as determined by FRAP, and R is the corrected concentration ratio after considering the effect of diffusion coefficient of NETs (see the previous functions and Table 1).

**Protein**	**Mobile (A)**	**Immobile (1-A)**	**τ½ (s)**
wt LBR	39±6%	61±6%	47.97
LBRΔ63-172	63±8%	37±8%	44.58
NET51	88±8%	12±8%	31.74
Nesperin-3α	39±7%	61±7%	50.82
ER	99±6%	1±6%	37.65

Supplementary Table 1. Fraction recovery as determined by fitting. The data from the FRAP experiments (Supplementary Fig 5.) was fit with the equation $I(t) = A(1 - e^{-\tau_1 t})$. The mobile (A) and immobile fractions (1-A) were determined by fitting with the aforementioned equation. The τ½ ($\tau_{1/2} = ln 0.5/\tau_1$) column represents the time at which half of the final fluorescence recovery occurred.
